# Mental health and conflict: a pilot of an online eye movement desensitisation and reprocessing (EMDR) intervention for forcibly displaced Syrian women

**DOI:** 10.3389/fpubh.2024.1295033

**Published:** 2024-05-30

**Authors:** Aseel Hamid, Amanda C. de C. Williams, Muslihah Albakri, Katrina Scior, Sian Morgan, Hamodi Kayal, Matthew Wilcockson, Rouba Drouish Alkaja, Sahbaa Alsayed, Robin Logie, Shiraz Farrand, Walid Abdul-Hamid

**Affiliations:** ^1^Research Department of Clinical, Educational and Health Psychology, University College London, London, United Kingdom; ^2^The Bridge Group, London, United Kingdom; ^3^Trauma Aid UK, Somerset, United Kingdom; ^4^Coventry University and Coventry and Warwickshire Partnership Trust, Coventry, United Kingdom; ^5^Independent Psychologist, Gaziantep, Türkiye; ^6^Central Team, Surrey, United Kingdom

**Keywords:** conflict, EMDR, mental health, refugee, intervention, Syria, trauma, PTSD

## Abstract

**Background:**

The Syrian conflict has been ongoing since 2011. Practical and scalable solutions are urgently needed to meet an increase in need for specialised psychological support for post-traumatic stress disorder given limited availability of clinicians. Training forcibly displaced Syrians with a mental health background to remotely deliver specialised interventions increases the availability of evidence based psychological support. Little is known about the effectiveness of online therapy for forcibly displaced Syrian women provided by forcibly displaced Syrian women therapists.

**Purpose:**

To pilot an evidence-based trauma therapy, Eye Movement Desensitisation and Reprocessing (EMDR), carried out online by trained forcibly displaced Syrian women therapists for forcibly displaced Syrian women who require treatment for post-traumatic stress disorder (PTSD).

**Methods:**

83 forcibly displaced Syrian women, living in Türkiye or inside Syria, with diagnosable PTSD, were offered up to 12 sessions of online EMDR over a period of 3 months. This was delivered by forcibly displaced Syrian women therapists who were trained in EMDR. Data were gathered, using Arabic versions, on PTSD symptoms using the Impact of Events Scale Revised, depression symptoms using the Patient Health Questionnaire-9 and anxiety symptoms using the Generalised Anxiety Disorder Assessment-7 at baseline, mid-point, and end of therapy.

**Results:**

PTSD scores, depression scores and anxiety scores all significantly reduced over the course of treatment, with lower scores at midpoint than baseline and lower scores at end of treatment than at midpoint. Only one participant (1%) exceeded the cutoff point for PTSD, and 13 (16%) exceeded the cutoff points for anxiety and depression at the end of treatment.

**Conclusion:**

In this pilot study up to 12 sessions of online EMDR were associated with reductions in PTSD, anxiety and depression symptoms in Syrian women affected by the Syrian conflict. The training of forcibly displaced Syrian mental health professionals to deliver online therapy is a relatively low cost, scalable, sustainable solution to ensure that those who are affected by the conflict can access specialised support. Further research is needed using a control group to confirm that the observed effects are due to EMDR treatment, as is research with post-treatment follow-up to ascertain that benefits are maintained.

## 1 Introduction

In Syria in 2011, a peaceful uprising was met with a cascade of increasingly violent government responses that have continued for over a decade (1). In 2023, the situation is even more complex, with various armed groups and foreign governments being party to the conflict, and it remains a cause of one of the largest displacement crises in the world (2). Inside Syria, 14.6 million people needed humanitarian assistance and over 12 million Syrians have been forced to move within or across borders, with 6.8 million internally displaced and 5.4 million living as refugees in Türkiye. Lebanon, Jordan, Iraq and Egypt (3). Forcibly displaced persons are those who are forced to move within or across borders and include refugees, asylum seekers and internally displaced persons. There is an increasing need for mental health and psychosocial support for forcibly displaced Syrians, exacerbated by limited legal and healthcare infrastructure of host nations, poverty, discrimination, rejection, violence from host communities and a general lack of Arabic speaking interpreters (4). The earthquake that devastated Türkiye and Syria in 2023 has caused further destruction with reduced international aid provision, further compounding the need for psychological support (5).

Practical and scalable solutions are urgently needed amidst limited availability of clinicians. As recommended in widely endorsed international guidelines on working in humanitarian disasters (6), training workers within the community to provide this support is a scalable, sustainable, and culturally sensitive way to reduce the treatment gap. Guidelines also highlight the importance of working with communities and influential community figures, at multiple levels of support, from ensuring basic safety and security all the way through to the provision of specialised psychological support (6, 7). Where specialised support, such as trauma-focused interventions, is required, recommendations involve the training of local community workers in evidence-based trauma therapies including EMDR and trauma-focused cognitive behavioural therapy (7, 8). Specialised support denotes the additional support required for populations who have significant difficulties in functioning and severe mental health problems, where their needs exceed the capacities of existing primary health services.

Whilst several studies have evaluated the effectiveness of scalable culturally adapted non-specialised interventions in forcibly displaced populations, such as Problem Management Plus (9, 10) and Culturally Adapted Cognitive Behavioural Therapy (11), very few studies have evaluated specialised mental health interventions. EMDR has been shown to be effective, both as individual therapy (12) and as group therapy (13) within forcibly displaced populations. EMDR is based on the idea that negative thoughts, feelings, and behaviours are a result of unprocessed memories and therapy involves standardised procedures that include focusing on spontaneous associations of traumatic images, thoughts, emotions and bodily sensations whilst simultaneously focusing on bilateral stimulation with the aim of processing memories (8). A systematic review and meta-analysis showed that EMDR effectively reduced PTSD, depression and anxiety symptoms compared to treatment as usual, CBT or wait-list, amongst global forcibly displaced populations (14). A systematic review and meta-analysis called for the expansion on the current evidence base on EMDR in forcibly displaced populations (15).

Given the exodus of healthcare workers during the conflict, there is a shortage of skilled professionals to provide culturally and faith-sensitive specialised support to the dispersed population of forcibly displaced Syrians, including internally displaced Syrians, Syrians in neighbouring countries such as Türkiye, Jordan, or Lebanon, as well as in Europe (16–18). Severe shortages of mental health professionals for internally displaced Syrians have sparked some initiatives to provide online training, education, and consultation for Syrian mental health professionals. A network of 20 mental health specialists offered online consultations to referrers, mainly for internally displaced Syrians, with positive feedback on its acceptability (19). In addition, Trauma Aid UK, a non-governmental organisation, used a hybrid model of training in Türkiye, where 86 clinicians, the majority of whom were forcibly displaced Syrians with a background in mental health, were provided with extensive EMDR training that led to accreditation (20, 21). Since the inception of Trauma Aid UK EMDR training, over 150 mental health professionals now work with refugees in Arab countries (21). In addition, trained clinicians are given the opportunity for ongoing remote EMDR supervision, and, over time, some clinicians have themselves become EMDR supervisors.

Forcibly displaced Syrian mental health professionals reported that their shared experiences of war and displacement with their Syrian clients, as well as their shared characteristics and language helped to build rapport, trust and understanding of their Syrian clients (22). Syrian cultural and social norms underlie a preference for gender-congruent mental health care (12, 22). This is consistent with previous literature, where gender and language differences were seen as barriers to receiving good quality care for Syrian refugees in Greece (18).

The provision of online mental health interventions can also increase access to and availability of psychological support across forcibly displaced populations. Systematic reviews of online mental health interventions show that they are cost-effective with high overall treatment satisfaction (23, 24). The COVID-19 pandemic further necessitated a permanent global increase of and reliance upon remote psychological support given its practical advantages. Guidance on using EMDR online has since been developed (25, 26) and online EMDR has shown to be acceptable to EMDR clients previously receiving EMDR in person, with 88% of 142 EMDR clients reporting that they were very or extremely comfortable with receiving therapy online (27).

Despite the global, post-pandemic, proliferation of online mental health, research on effectiveness is still in its infancy. A systematic review investigating the effects of online EMDR (28) found only one eligible trial showing that combined internet delivered CBT and EMDR successfully reduced self-rated and clinician-rated PTSD from baseline to follow-up (29). A handful of studies exist on the effectiveness of online EMDR, and findings show clinically meaningful reductions in PTSD, depression, and anxiety (30) and similar outcomes, based on subjective units of distress (SUDs), compared to previous EMDR studies delivered face-to-face (31).

Less is known about the effectiveness of online psychological interventions for forcibly displaced populations. In a mixed methods single-arm feasibility trial, eight sessions of a remote group parenting intervention and group, EMDR were provided to displaced asylum seekers in the UK. Participants noted that video sessions in their own homes felt comfortable and gave them privacy and distance from others’ judgements (32). Working virtually can reduce physical and attitudinal barriers to care, overcome barriers of lack of privacy, space and perceived confidentiality concerns reported in clinics where Syrians are forcibly displaced (18), whilst increasing available resources.

Armed conflict and forcible displacement create significant risks of women being exposed to increased threats and violence (33). Marital violence increases during times of war because social isolation and loss of support systems enable domestic violence and humiliation (34). War and forcible displacement have been shown to strengthen patriarchal structures (35), and Syrian women have been reported to experience significant additional restrictions to their lives and freedom (36). At the same time, conflict has contributed to changing social norms and expectations and women have often become key agents in healthcare provision, particularly inside Syria where there is a shortage of health professionals (37).

It is not surprising, then, that forcibly displaced Syrians were shown to have prevalence rates of symptoms of anxiety, depression, and PTSD of 36.1%, 34.7%, and 19.6%, respectively (38). In addition, women were twice as likely to experience PTSD and depression than their male counterparts, after adjusting for post-displacement stressors, potential trauma exposure and sociodemographic characteristics (38). The experience of sexual violence increases reluctance to seek help. Reported barriers to disclosure of distress include fears of being stigmatised (39, 40), fears around consequences of disclosure, the absence of social support, and distrust of health professionals (41).

Trauma focused interventions for Syrian women should be culture- and gender-sensitive (40). This study is the first to evaluate online EMDR for forcibly displaced Syrians. We aimed to evaluate the preliminary outcomes of an economical approach to provide a gender and culturally sensitive intervention for a neglected population. Specific hypotheses were formulated:

*Hypothesis 1*: There will be a significant decrease in depression, anxiety, and PTSD scores following the intervention.

*Hypothesis 2*: Socio-demographic characteristics such as host location, age and support network would be linked to depression, anxiety, and PTSD scores at baseline and following the intervention.

*Hypothesis 3*: Experience of trauma in childhood, particularly repeated childhood trauma, would be linked to higher scores of depression, anxiety and PTSD at baseline and at end of treatment.

## 2 Materials and methods

### 2.1 Participants and procedures

This observational pilot study took place between November 2020 and December 2021; all research meetings, clinical supervision, eligibility sessions and intervention sessions were conducted online. Three female Syrian psychologists who had been forcibly displaced to Southern Türkiye, and were trained in EMDR, were recruited to provide up to 12 sessions of EMDR. Each therapist was allocated a clinical supervisor with extensive EMDR experience. Two of the three supervisors were native Arabic speakers, and one therapist was proficient in English. Therapists were required to collect data using a secure online form developed for the study. Given the political sensitivities of recording sessions to check fidelity, supervisors instead regularly checked in with therapists on fidelity of the sessions with therapists using process notes and therapists’ verbal accounts. The therapists conducted the standard EMDR protocol over 12 sessions, adapted for language as the therapy was conducted exclusively in Arabic. The EMDR protocol, which also included a preparation phase consisting of stabilisation exercises, was administered online using videoconferencing functions on Zoom and WhatsApp, using eye movements for bilateral stimulation.

NGOs working with forcibly displaced Syrians were approached, and a leaflet on the project and information sheets were provided to advertise the project. All NGOs accepted advertising this in their offices and virtually, and they were chosen based on the network of the Syrian therapists and researchers. Inclusion criteria were being female, Syrian, aged between 18 and 60 years, forcibly displaced by the Syrian conflict, and experiencing PTSD symptoms (using cut-off points as described below). Availability of resources limited sessions to 12, and exclusion criteria included where moderate or high dissociation was indicated by a total score of 9 on the Brief Dissociative Experiences Scale (42), symptoms of psychosis, or reported suicide attempts during the initial recruitment session, for which women were signposted to relevant services.

**Figure 1:**
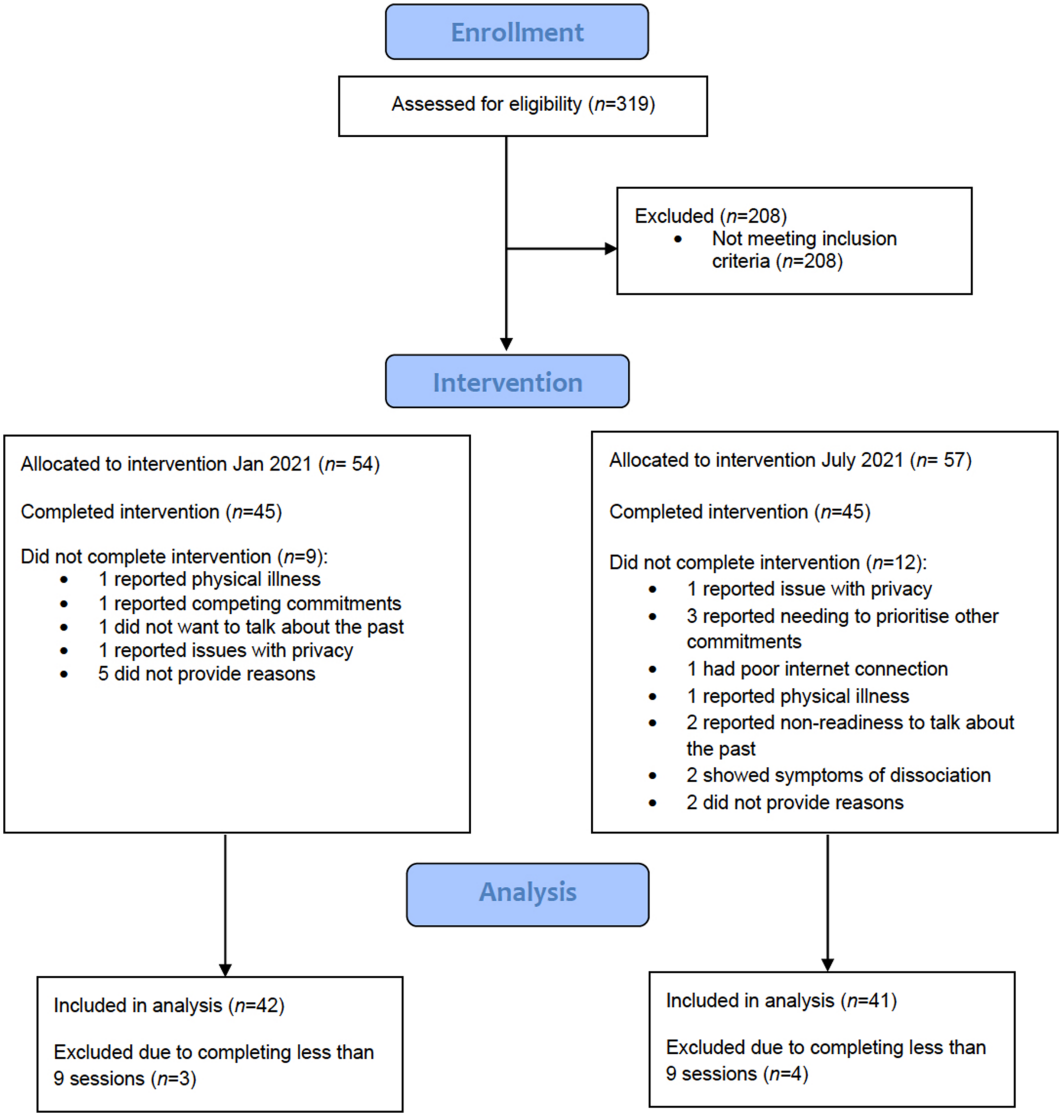
Participant flow diagram.

A total of 319 participants were assessed for eligibility to take part in the intervention during a one hour long online video call by one of the therapists. Informed consent was sought from all participants. Figure 1 presents a participant flow diagram showing participants were recruited at two timepoints (January 2021, *N* = 45, July 2021, *N* = 45). In total, 83 participants completed online EMDR. There is an absence in the research literature on the minimum number of sessions of EMDR attended to be classified as complete (43) and guidance on EMDR for adults outlines that 8 to 12 sessions are typically provided (44). Consequently, we required that participants completed at a minimum of nine (out of a total of 12) sessions and to have completed outcome measures at two or more timepoints to be considered to have completed treatment. We decided to use nine sessions instead of eight sessions as a cut-off point to reflect greater severity of difficulty and therefore greater need for sessions. Accordingly, a total of seven participants were excluded from the analysis due to completing less than nine sessions; these participants had come to a shared agreement with their therapist that they had reached recovery and completed their treatment goals and did not need to attend their remaining three sessions. Where provided, reasons for non-completion included poor internet connection, issues with privacy, physical illness, caring responsibilities and needing to prioritise other commitments.

### 2.2 Measures

**Figure 2:**
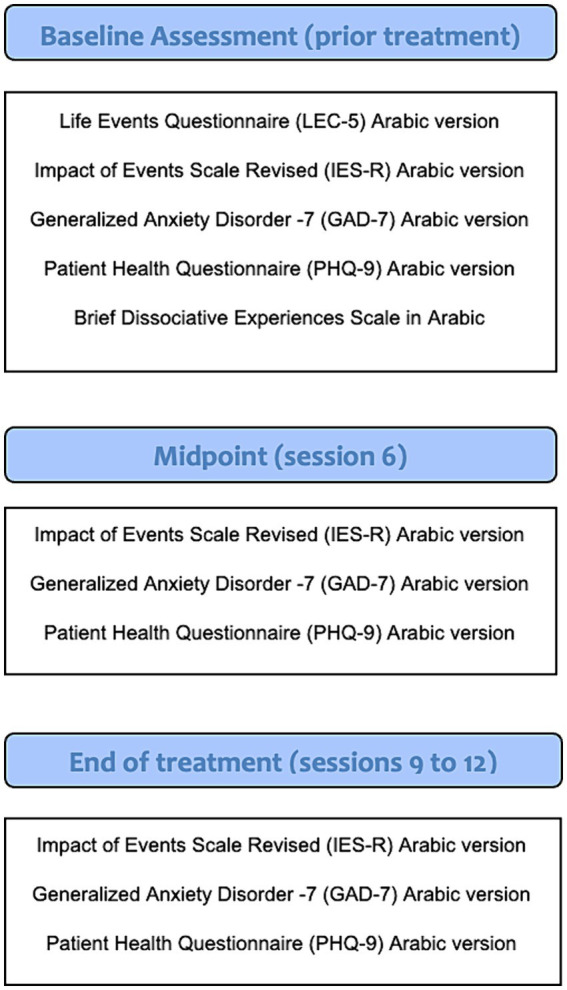
Measures used throughout study phases.

Outcome measures were administered at three time points: assessment (baseline), midpoint of treatment, and end of treatment; see Figure 2 for a visual representation of the measures conducted during each phase.

#### 2.2.1 Life Events Checklist 5

The Life Events Checklist 5 (LEC-5) evaluates respondents’ exposure to a wide range of stressful and traumatic experiences (45). Participants responded to 16 stressful events known to potentially result in distress by checking items. The scale ranges from *happened to me*, *witnessed it*, to *learned about it*, with additional options of *not sure*, and *does not apply*. An Arabic version of the LEC-5 administered to Palestinian refugees in Lebanon had acceptable reliability, with a Cronbach’s alpha of 0.71 (46). The Arabic version of the LEC-5 was administered, and the purpose of this measure was to gather assessment data and was administered at baseline only.

#### 2.2.2 Impact of events scale revised

The impact of events scale revised (IES-R) (47) was used to assess PTSD symptoms. The IES-R is a 22-item tool commonly used clinically and in research to assess posttraumatic stress by self-report, with subscales for intrusion/reexperiencing, hyperarousal, and avoidance. Item responses range from 0 (*not at all*) to 4 (*extremely*), with a maximum score of 88, with higher scores indicating higher levels of PTSD symptoms. Although not used for the diagnosis of PTSD, a cutoff score of 33 has been used to discriminate between those with and without PTSD (48, 49). We used the cutoff of 33 in this research.

The IES-R has a well-established Arabic version developed for use initially with Middle Eastern refugees in Australia (50) with good discriminant validity and reliability. It has since been used with forcibly displaced Syrians (51, 52) and has shown high internal consistency, with a Cronbach’s alpha of 0.8 or above (12, 13, 53). This Arabic version of the IES-R was administered at baseline, midpoint and end of therapy.

#### 2.2.3 Patient health questionnaire 9

The patient health questionnaire 9 (PHQ-9) is a nine-item measure of depression symptoms (54); items are rated for frequency using a scale from 0 (*not at all*) up to 3 (*nearly every day*), yielding a total severity score between 0 and 27. A meta-analysis concluded that the cut-off scores between 8 and 11 can detect major depressive disorder (55). In this study, we used the more conservative cut-off score of 8.

Arabic versions of the PHQ-9 have been reported to be internally consistent, with Cronbach’s alpha of 0.8 or above in a Lebanese population (56) and a Saudi population (57). PHQ-9-was administered at baseline, midpoint and end of therapy in Arabic.

#### 2.2.4 Generalised anxiety disorder-7

The generalised anxiety disorder-7 (GAD-7) is a seven-item measure of anxiety symptoms (58); it is rated in the same way as the PHQ-9 yielding a total severity score between 0 and 21. The recommended cut-off score for the GAD-7 ranges from 7 to 11 (59). In this study, we have used the more conservative cut-off score of 7.

The Arabic version of the GAD-7 was shown to be highly reliable, with a Cronbach’s alpha of above 0.9 (56). This Arabic version of GAD-7 was administered at baseline, midpoint and end of therapy in Arabic.

#### 2.2.5 Impact of displacement, trauma, and violence

To gather further data at initial assessment, participants completed short questions to gauge the impact of sociodemographic and post-displacement context on their wellbeing. In addition, participants were asked to rate the impact that different types of gender-based violence that they may have experienced (emotional, physical, sexual, financial, early marriage, forced marriage), has had on their mental health problems on a Likert scale from 1 to 10. These questions were selected given that ongoing post migration stressors have been found to significantly mediate the effect of war exposure and mental health problems in forcibly displaced populations (60, 61) and that financial difficulties and a lack of social support predicted mental health problems in a sample of forcibly displaced Syrians in Türkiye (38). In addition, data was gathered on whether a participant experienced childhood trauma, and whether this was repeated trauma, as research has shown that childhood onset trauma has been associated with poorer treatment outcomes (62).

### 2.3 Data analysis

To address missing data within the IES, GAD-7 and PHQ-9 scales, mean scores were imputed (no more than one missing value for GAD-7 and PHQ-9 scales respectively, and no more than two missing values for the IES scale for each participant). Where midpoint data was available but not endpoint (*n* = 4), mean midpoint scores on the IES, GAD-7 and PHQ-9 scores were carried forward to endpoint.

*Hypothesis 1*: Since the data violated the Shapiro-Wilks normality test, Friedman, and Wilcoxon post-hoc tests were used to compare the mean scores of the psychometric scales across the three timepoints. A Bonferroni correction for multiple comparisons was applied.

*Hypothesis 2*: To determine whether socio-demographic factors including host location, age, education and social support network were linked to depression, anxiety, and PTSD scores, Spearman’s correlations, Mann–Whitney tests, and Kruskal-Wallis tests were used. A Bonferroni correction for multiple comparisons was applied.

*Hypothesis 3*: Mann–Whitney tests were used to examine whether the experience of childhood trauma and the experience of repeated trauma were linked to higher depression, anxiety, or PTSD scores.

All analyses were performed in IBM SPSS software version 25. An *a priori* power analysis was undertaken using G*Power 3.1 (63) for a repeated measures ANOVA, within factors (1 group, 3 measurements) with a small effect size (*F* = 0.3) and alpha of 0.05. A sample size of 20 was necessary at each timepoint to achieve a power of 0.8.

## 3 Results

### 3.1 Baseline characteristics

Figure 1 shows the flow of participants and Table 1 shows the demographic characteristics of included participants.

**Table 1:** Demographic characteristics of participants.

Variable	Mean	*n*
**Marital status**		
Single	15%	12
Married	51%	42
Divorced	15%	12
Widowed	20%	16
**Highest education qualification**		
Primary level or lower	16%	13
GCSE equivalent	10%	8
A level or equivalent	28%	23
Degree or higher	46%	38
**Employment status**		
Employed	66%	55
Unemployed	28%	23
Volunteering or studying	6%	5
**Visited a mental health professional in Syria prior to 2011**		
Yes	23%	19
No	77%	63
**Self-rated physical health**		
Requires medical attention	17%	14
Good	60%	49
Very good/excellent	22%	18

Of the 83 participants who completed treatment, most participants identified as Syrian Arab (*n* = 77), two as Turkmani, one as both Syrian and Turkmani, one as Circassian, one as Kurdish and one participant not providing data on ethnicity. Participants had a mean age of 33.2 years, with a range of 19 years to 52 years. The mean number of children participants had was 2, ranging from 0 to 5 children. Most participants (77%) had not visited a mental health professional in Syria prior to 2011. Participants were based in a range of countries including Turkey (48%), Syria (26%), and Germany (6%).

Table 2 presents the contextual characteristics of participants including their social support and life events checklist.

**Table 2:** Contextual characteristics of participants.

Variable	%	*n*
**Social support network size**		
Zero	12%	10
1 to 5 people	80%	66
6 to 9 people	7%	6
10 or more	1%	1
**Self-rated impact of COVID-19**		
No impact	4%	3
Little impact	24%	20
Strong impact	72%	59
**Self-rated impact of daily environmental stressors**		
No impact	27%	22
Little impact	49%	40
Strong impact	24%	20
**Life events checklist (percentage selecting ‘happened to them’ or ‘witnessed event’)**		
Natural disaster	45%	37
Fire or explosion	71%	58
Transportation accident	43%	35
Serious accident at work, home, during recreational activity	45%	37
Exposure to toxic substances	20%	16
Physical assault	71%	58
Assault with a weapon	53%	42
Sexual assault	51%	42
Other unwanted or uncomfortable sexual experience	39%	32
Combat or exposure to warzone	89%	73
Captivity	44%	36
Life-threatening illness or injury	41%	34
Severe human suffering	85%	69
Sudden violent death	43%	35
Sudden unexpected death of a loved one	65%	53
Causing serious injury or harm to someone else	21%	17
Any other very stressful event	83%	67

### 3.2 Treatment outcome by recruitment periods

An independent sample t-test was conducted to determine whether there were any differences in participants recruited at different time points at baseline assessment and at the end of treatment. At baseline assessment, there were no statistically significant differences in mean age or number of children, marital status, or current employment status between the two groups. Those recruited in January 2021 had higher IES mean scores at baseline assessment (*M* = 61.4) than those recruited in July 2021 (*M* = 56.1), *p* = 0.04. At the end of therapy, participants recruited in July 2021 had significantly lower anxiety scores (*M* = 2.78) than those recruited in January 2021 (*M* = 4.33), *p* = 0.01. There were no observed differences in depression scores at baseline nor at endpoint (*p* > 0.05).

### 3.3 Hypothesis 1: intervention and symptom reduction

Friedman test by ranks revealed statistically significant differences in PTSD, depression and anxiety scores across the three timepoints, showing support for hypothesis 1. For PTSD, baseline (*mdn* = 60), midpoint (*mdn* = 20) and endpoint (*mdn* = 4), χ^2^(2) = 146.18, *p* < 0.001. For depression, baseline (*mdn* = 16), midpoint (*mdn* = 10) and endpoint (*mdn* = 4), χ^2^(2) = 122.41, *p* = 0.000. For anxiety, baseline (*mdn* = 15), midpoint (*mdn* = 7) and endpoint (*mdn* = 3), χ^2^(2) = 129.41, *p* < 0.001. This suggests that online EMDR therapy was beginning to show its impact at midpoint, i.e., after six sessions, at midpoint.

*Post hoc* analysis using Wilcoxon signed rank tests showed PTSD, depression, and anxiety scores were significantly lower at endpoint than at midpoint and at baseline. PTSD, depression, and anxiety scores at midpoint were also significantly lower than at baseline (all *p* < 0.001 with Bonferroni correction). Table 3 shows the statistics of the *post hoc* tests.

**Table 3:** Friedman’s test *post hoc* analysis using Wilcoxon signed rank tests (with Bonferroni correction).

Friedman’s test by ranks	Test statistic	Df	Sig
GAD at baseline, midpoint and endpoint	129.41	2	0.00
	**Mean rank**		
GAD at baseline	2.91		
GAD at midpoint	1.95		
GAD at endpoint	1.14		
	**Test statistic**	**SE**	**Adj sig**
GAD at endpoint—GAD at midpoint	0.81	0.16	0.00
GAD at endpoint—GAD at baseline	1.76	0.16	0.00
GAD at midpoint—GAD at baseline	0.96	0.16	0.00
	**Test statistic**	**Df**	**Sig**
PHQ at baseline, midpoint and endpoint	122.41	2.00	0.00
	**Mean rank**		
PHQ at baseline	2.88		
PHQ at midpoint	1.98		
PHQ at endpoint	1.14		
	**Test statistic**	**SE**	**Adj sig**
PHQ at endpoint—PHQ at midpoint	0.84	0.16	0.00
PHQ at endpoint—PHQ at baseline	1.74	0.16	0.00
PHQ at midpoint—PHQ at baseline	0.90	0.16	0.00
	**Test statistic**	**Df**	**Sig**
IES at baseline, midpoint and endpoint	146.18	2.00	0.00
	**Mean rank**		
IES at baseline	2.96		
IES at midpoint	1.99		
IES at endpoint	1.05		
	**Test statistic**	**SE**	**Adj sig**
IES at endpoint—IES at midpoint	0.94	0.16	0.00
IES at endpoint—IES at baseline	1.91	0.16	0.00
IES at midpoint—IES at baseline	0.98	0.16	0.00

Table 4 shows the percentage of participants who exceeded cutoff scores based on PTSD, depression, and anxiety across the three timepoints. A Reliable Change Index (RCI; 64) was calculated for each participant, comparing baseline and endpoint scores for PTSD, anxiety, and depression, using Morley and Dowzer’s (65) RCI calculator. All (100%) of participants showed a reliable improvement in PTSD scores. Similarly, almost all participants reliably improved in depression (93%) and anxiety (91%) at the end of therapy.

**Table 4:** Percentage of participants who were symptomatic at three timepoints.

Measure	Assessment *n* (%)	Midpoint *n* (%)	Endpoint *n* (%)
IES	82 (100%)	14 (18%)	1 (1%)
PHQ-9	78 (95%)	50 (63%)	13 (16%)
GAD-7	77 (94%)	45 (56%)	13 (16%)

### 3.4 Hypothesis 2: sociodemographic characteristics and symptom scores

There was some support for hypothesis 2, that participants’ host location was linked to symptom scores as participants based outside of Syria had significantly higher anxiety scores than those in Syria at baseline (*U* = 313, *p* > 0.05). At the end of therapy, participants in Syria had significantly higher PTSD scores than participants outside of Syria (*U* = 366, *p* = 0.02).

However, socio-demographic characteristics including age, highest education qualification, current employment status, marital status, and social support network size were not significantly associated with PTSD, depression, or anxiety scores at baseline, nor at the end of therapy. Self-rated physical health and a history of poor mental health were also not significantly related to mental health scores (*p* > 0.05).

### 3.5 Hypothesis 3: childhood trauma and symptom scores

To test hypothesis 3, that experience of trauma in childhood would be linked to higher depression, anxiety and PTSD scores at baseline and following the intervention, a Mann–Whitney test was conducted. Findings showed that participants who experienced childhood trauma had significantly higher PTSD scores following therapy than those without childhood trauma (*U* = 281, *p* = 0.03). However, this difference was not observed for anxiety (anxiety: *U* = 429, *p* > 0.05), or for depression (*U* = 355, *p* > 0.05), nor for any of the scores at baseline (anxiety: *U* = 415, *p* > 0.05; depression: *U* = 346, *p* > 0.05). Repeated childhood trauma was linked with significantly higher depression both at baseline (*U* = 585.0, *p* = 0.026) and following therapy (*U* = 569.5, *p* = 0.01), but not for PTSD or anxiety scores at baseline (PTSD: *U* = 684, *p* > 0.05; anxiety: *U* = 807.5, *p* > 0.05) or end of treatment (PTSD: *U* = 642, *p* > 0.05; anxiety: *U* = 785, *p* > 0.05).

### 3.6 Post-hoc tests on impact of abuse

We also conducted Spearman’s correlations to investigate whether there was a link between self-rated impact of different types of abuse and scores of anxiety, depression and PTSD at the beginning and end of therapy. Self-rated impact of financial abuse was significantly correlated with depression scores (*r* = 0.40, *p* < 0.001) and anxiety scores (*r* = 0.31, *p* < 0.001) at baseline, in which greater perceived financial abuse was linked to greater symptoms. This difference was no longer significant at the end of treatment (depression: *r* = 0.19., *p* > 0.05; anxiety: *r* = −0.28., *p* > 0.05). The impact of other forms of abuse including physical, psychological, and sexual was not significantly associated with mental health scores at baseline, nor at end of treatment.

## 4 Discussion

This investigation has shown that online EMDR appears to be effective in reducing symptoms of PTSD, depression, and anxiety at the end of up to 12 sessions, even when using conservative cut-off points. This conclusion should be tested in a controlled study to rule out the passage of time since trauma was experienced or treatment effects not specific to EMDR.

Consistent with research suggesting that childhood onset trauma is associated with poorer treatment outcomes (62), participants who reported that they experienced childhood trauma had higher mean PTSD scores at end of treatment than women who did not report experiencing childhood trauma. Those with repeated childhood trauma had significantly higher depression scores both at baseline and end of treatment. Despite this, online EMDR remained effective in significantly reducing symptoms for all three outcomes. This may suggest that those who have experienced childhood trauma, particularly repeated childhood trauma, may benefit from more than 12 sessions of online EMDR to further increase treatment gains. This is in line suggestions that at least 20 sessions are needed to achieve comprehensive improvements in those who have experienced repeated childhood trauma (66), although more research needs to be conducted to reach a consensus (67).

Given the importance of daily stressors on the mental health of conflict-affected populations (60, 61) and the continued violence in Syria (2), it was unsurprising that internally displaced Syrian women showed significantly higher PTSD levels at the end of therapy than women displaced outside of Syria. Despite this, all Syrian women showed a reliable improvement in PTSD scores at the end of therapy, suggesting that EMDR may be effective in situations of ongoing trauma.

The training and supervision of forcibly displaced Syrian mental health professionals to provide EMDR to Syrians online is a promising and relatively low-cost initiative that can promote capacity building and sustainably reduce the treatment gap in this population, which has further widened since the earthquakes in Syria and Türkiye in 2023 (68). The Eastern Mediterranean region has recently been disproportionately affected by multiple, ongoing emergencies leading to loss, destruction and trauma. For example, as of 2024, around 25 million people in Sudan require humanitarian assistance due to the ongoing civil war (69) and over 2 million people require humanitarian assistance in Gaza due to Israeli military hostilities (70). In 2023, 300,000 people required humanitarian assistance due to the earthquakes in Morocco (71), over 880,000 required assistance due to the cyclone in Libya (72). This further necessitates the availability of evidence-based interventions in the region.

Self-rated impact of financial abuse was significantly correlated with depression scores at the end of treatment, in line with previous literature that financial abuse predicted mental health problems in forcibly displaced Syrians in Türkiye (38). Previous research on Syrian women in Jordan found that women whose financial dependence on their husbands either increased or decreased since the conflict were more likely to experience intimate partner violence (73) suggesting that fundamental tensions and shifts in the structure of financial responsibilities due to poverty, displacement and changing gender roles underpin violence. In addition, Syrian women who are financially dependent on their husbands are often unable to escape financial or other forms of abuse due to fears around their and their children’s financial wellbeing (74).

Whilst the results of this pilot study suggest that EMDR appears to be effective, some important considerations regarding the provision of online support to a forcibly displaced population are discussed in turn below.

As the practice of online mental health provision grows, there are increasing recommendations and guidance on conducting EMDR remotely. The overall recommendation is to follow the standard protocol as one would in face to face and not to alter the protocol when working in an online setting (26).

EMDR training guides clinicians to be attentive to clients’ safety and comfort levels during therapy. Delivering EMDR online may increase the risk for misattunement between the client and the therapist due to, e.g., the possibility of technical glitches, limited eye contact, limited cues and the absence of an in-person connection (75). Therapists using EMDR online should be trained using existing guidance on establishing safety and maintaining the therapeutic relationship remotely, and ways to manage complications and unpredictability that may come with conducting EMDR remotely (25).

Additionally, a safe and comfortable environment is vital for clients to be able to engage in therapy. However, a therapist does not have sufficient control over participants’ environment during online therapy. This is an important consideration given that most clients engage in online therapy in their own homes. In some cases, as with all online mental health support, the provision of online EMDR at home can lead to greater feelings of privacy and confidentiality (32). However, for survivors of violence and abuse perpetrated in the home, this would at best lead to privacy issues leading to a lack of full engagement from the clients’ part due to concerns about being overheard, as was reported in three cases during this study which led to disengagement. At worst, this would place survivors at risk of further violence from perpetrators. Practical measures can help to reduce the risks of violence faced by Syrian women, such as the provision of safe forms of transport and community “safe spaces” (76); these may also be used to discreetly provide a confidential space to partake in online therapy.

Whilst online delivery and training increase resources, they also may make it more difficult to coordinate health responses. Syrian health care workers have historically faced multiple barriers to providing care, including a fragmented response, politicisation of health care support, attacks on health care workers particularly inside Syria, a lack of access to advanced specialty training and difficulties confirming staff credentials (77, 78). In the case of the provision of specialised mental health services, it is crucial to ensure a coordinated online response, and a hybrid support system based on the clients’ location. The integration of specialised online support with local services is key, particularly to ensure that any issues of risk are sufficiently dealt with.

The experiences and characteristics that forcibly displaced Syrian women therapists share with their clients can overcome the language and gender differences that may act as barriers to seeking and engaging with support (18). It provides a gendered and cultural innovation, which is seen to be key to ensure acceptability and higher access and engagement in online mental health support for Syrians (4). In a qualitative study with forcibly displaced Syrian mental health professionals providing support to Syrians within the community, all had noted that their shared reality of war and displacement enabled better understanding and trust within therapy and used their nuanced understanding of their shared language and culture as a tool (22). A smaller proportion noted that their shared experiences often led to unpleasant reminders of their own traumatic experiences, highlighting the importance of using peer supervision and personal therapy as a means of coping. In addition, Syrian mental health professionals exhibited higher levels of secondary traumatic stress as well as higher levels of satisfaction from providing care compared to other therapists globally (79). Taken together, this highlights the ethical imperative of ongoing clinical supervision in humanitarian contexts even though this is often deprioritised and underresourced. However, initiatives to provide clinical supervision in Türkiye emphasise that supervision is contextualised and considers sociocultural factors (80), and the integrated model for supervision handbook provides general principles and best practice (81).

Some noteworthy limitations of this study need to be acknowledged. This study had a relatively small sample; whilst adequate for a pilot study, caution needs to be exercised in generalising the results to the wider forcibly displaced Syrian population. This relatively small sample also meant that we were unable to assess for the presence of therapist effects on the treatment. Whilst the RCIs showed reliable improvement for PTSD scores, further investigation of clinically significant change (65) was beyond the scope of the study but would have been useful. In addition, due to limited resources, there was no control group. Without a control group and randomisation to groups, it is difficult to attribute the observed effects to the intervention alone. Also, participants were not followed up some time after the end of treatment to determine whether treatment effects were maintained. This is important as there is not enough evidence on whether the effects of trauma-focussed interventions are maintained (82).

This initial research maps onto various priorities identified in the Interagency Standing Committee Consensus-Based Research Agenda for mental health and psychosocial support in humanitarian settings (83), including assessing the outcomes and impact of interventions, the effectiveness for remote and digital MHPSS interventions and ensuring the sustainability of interventions. Given the symptom reduction seen in this pilot study, future research should investigate the effectiveness of online EMDR for forcibly displaced Syrians using randomised controlled trials with a follow up. This should also include a qualitative component from both the perspectives of Syrian forcibly displaced clients and Syrian forcibly displaced therapists, to ascertain the acceptability of this intervention, and to determine preferences, customisations, and adaptations that may be required to reduce access and engagement barriers and to facilitate uptake of this promising intervention. Mapping onto another research priority outlined in consensus (83), there is a need for a more in-depth investigation of the existing supervision models and strategies used by mental health professionals providing support for the Syrian population, drawing on existing guidance (81).

In line with guidelines and research, this should be investigated as part of an integrated, multidisciplinary, multi-systemic approach to recovery, that addresses both past trauma and the social determinants of refugee health (84, 85), whilst promoting community resilience (86). There is an increasing recognition of the adverse psychological impact of post-migration stressors, with therapy increasingly being complemented by case management aimed at helping forcibly displaced clients address social, legal, financial, housing, education, and employment issues (61). The pragmatic advantages of the provision of online therapy should therefore be complemented by a hybrid approach, where this specialised intervention is embedded within the healthcare system that forcibly displaced Syrians are residing in, to allow for the signposting and linking to existing services to manage postmigration stressors (84).

Some existing research with Syrian women has highlighted that the Syrian conflict may open social spaces to shift gendered norms and power dynamics in research, practice, and policy (37). Indeed, Syrian women who are trained to provide specialised support are fundamental actors to service the population of Syrian women who require this support, given the current strong preference for gender congruent care in this population.

## 5 Conclusion

The present research offers support for the benefits of online EMDR delivered by trained Syrian forcibly displaced women in reducing PTSD, depression and anxiety in forcibly displaced Syrian women. Online EMDR may offer a relatively low cost, scalable psychological intervention to address symptoms of PTSD, depression and anxiety.

## Data availability statement

The raw data supporting the conclusions of this article will be made available by the authors, without undue reservation.

## Ethics statement

The studies involving humans were approved by Research Department of Clinical, Educational and Health Psychology Ethics Chair (CEHP/2020/584) at University College London, United Kingdom. The studies were conducted in accordance with the local legislation and institutional requirements. The participants provided their written informed consent to participate in this study.

## Author contributions

AH: Conceptualization, Data curation, Formal analysis, Investigation, Methodology, Supervision, Project administration, Resources, Validation, Visualization, Writing – original draft, Writing – review & editing. AW: Writing – review & editing, Formal analysis, Methodology, Supervision, Validation. MA: Writing – review & editing, Data curation, Formal analysis, Software, Validation, Visualization, Writing – original draft. KS: Writing – review & editing. SM: Methodology, Supervision, Writing – review & editing, Conceptualization, Funding acquisition, Project administration. HK: Supervision, Writing – review & editing. MW: Supervision, Writing – review & editing. RD: Investigation, Writing – review & editing. SA: Investigation, Writing – review & editing. RL: Funding acquisition, Writing – review & editing. SF: Funding acquisition, Writing – review & editing. WA-H: Conceptualization, Funding acquisition, Methodology, Project administration, Resources, Supervision, Writing – original draft, Writing – review & editing.
